# 

**Published:** 1895-02

**Authors:** 


### TABLE OF CONTENTS.

ORIGINAL COMMUNICATIONS. PAGE

An Electrical Disinfectant. By ALBERT S. WOOLF, New York . . . . . 65

Sulphuric Acid and Peroxide of Sodium in the Treatment of Pulpless Teeth. By F. T. VAN WOERT, M.D.S., Brooklyn, N. Y. . . . . . . . . . . . 74

Note on Hydrogen Dioxide as furnished to Practising Dentists. By HENRY LEFFMANN, M.D., D.D.S. . . . . . . . . . . . . . . . . . . . . . . . 78

The Correction of Injury resulting from Extraction. By S. E. DAVENPORT, D.D.S., M.D.S. . . . . . . . . . . . . . . . . . . . . . . . . . 81

REPORTS OF SOCIETY MEETINGS.

New York Odontological Society . . . . . . . . . . . . . . . . . . . . . 84

Odontological Society of Pennsylvania . . . . . . . . . . . . . . . . . . 100

Harvard Odontological Society . . . . . . . . . . . . . . . . . . . . . . 104

Central Dental Association of Northern New Jersey . . . . . . . . . . . . 117

EDITORIAL.

Politics in Dental Associations . . . . . . . . . . . . . . . . . . . . . 124

The Dental Digest . . . . . . . . . . . . . . . . . . . . . . . . . . . 127

The Souvenir Volume of the Wells Celebration . . . . . . . . . . . . . . 127

Proof-Reader gone Wrong . . . . . . . . . . . . . . . . . . . . . . . . 127

DOMESTIC CORRESPONDENCE.

Reply to Dr. Max Greenbaum . . . . . . . . . . . . . . . . . . . . . . 128

Additions to Dr. Potter's Paper . . . . . . . . . . . . . . . . . . . . . 131

CURRENT NEWS.

Souvenir Volume of the Wells Celebration . . . . . . . . . . . . . . . . 131

SELECTIONS.

A New Method of reducing Dislocation of the Jaw . . . . . . . . . . . . 132

